# Problem-Based versus Lecture-Based Method in Pre-hospital Trauma Life Support Training; a Pre-test Post-test Study

**Published:** 2019-11-24

**Authors:** Masoumeh Falaki, Rouzbeh Rajaei Ghafouri, Samad Shams Vahdati

**Affiliations:** 1 Emergency Medicine Department, Faculty of Medicine, Tabriz University of Medical Sciences, Tabriz, Iran.; 2 Emergency Medicine Research Team, Tabriz University of Medical Sciences, Tabriz, Iran.

**Keywords:** Multiple Trauma, Emergency Medical Services, Problem-based Learning, Education

## Introduction

### Introduction:

Pre-hospital trauma life support (PHTLS) training is necessary for all emergency medical services (EMS) personnel to increase their efficacy and skills. This study aimed to compare two methods of problem-based learning (PBL) and lecture-based learning (LBL) for PHTLS training courses of EMS personnel. 

### Methods:

In this pre-post-test study, 144 male EMS staff members were divided into two groups of PBL (n=72) and LBL (n=72). Both groups received four sessions of PHTLS training based on 8^th^ edition of PHTLS guideline. The participants’ knowledge and skills were evaluated before and three months after training and the two groups were compared in this regard. 

### Results:

 The mean knowledge score (63.59±13.43 to 81.08±4.66; p<0.001) and mean skills score (58.85±19.74 to 99.07±25.02; p<0.001) of participants had significantly improved after the training courses. Both groups had similar scores before intervention, but PBL group had significantly higher scores in knowledge (p<0.001) and skills (p<0.001) after intervention. There was also a significantly higher change in knowledge (p<0.01) and skills (p<0.001) in PBL group compared to LBL group.

### Conclusions:

PHTLS training improves EMS personnel’s knowledge and skill in managing trauma patients. PBL was more effective than LBL.

## Introduction:

Taking care of the critically injured begins well before the patient arrives at a large academic trauma center. Pre-hospital care is the first part of the trauma treatment and care system (1). Pre-hospital trauma life support (PHTLS) plays a very important role in the effective management of trauma patients and aims to provide high-quality care that reduces injury and mortality in these patients (2, 3). The success of the pre-hospital care depends on the knowledge and skill of emergency medical services (EMS) personnel, which lead to improvements in the response time, interventions and, ultimately, the rapid transfer to the hospital (4-6).

EMS personnel are the first ones to examine and treat pre-hospital critical patients. It is important to increase the accuracy and speed of treatment to lower the mortality caused by these diseases (7). For this purpose, we need efficient and operational personnel to deal with various types of crashes and diseases (8, 9).

It is important to increase the staff’s awareness regarding PHTLS to decrease the mortality rate and increase their confidence and knowledge (10). A recent study has reported that after PHTLS training, the mortality rate had decreased (11). There is a need to improve EMS staff’s knowledge and attitude regarding PHTLS. 

Primary trauma care courses can provide the basic knowledge and skills necessary for identification and treatment of trauma patients in need of immediate resuscitation and stabilization of the injuries. These courses can teach an acceptable method for treating patients affected by trauma (5, 12). There are two common methods used for training, lecture-based learning (LBL) and problem solving methods (13). Problem-based learning (PBL) was implemented in the 1960s, which is now found in many training settings including medical sciences. PBL is an educational method focused on self-directed learning and small group discussions, which can provide learners with more opportunities for application of knowledge compared to traditional LBL method. Nowadays experts believe that PBL has many advantages over LBL, including flexible knowledge, promotion of communications and collaborative skills, and self-directed learning skills in a motivating format (14). 

Trauma care learning needs both knowledge and skill improvement, so LBL alone may not enhance trauma management skills. In this study, we compared the two methods of LBL and problem based method in improving knowledge and skills of EMS personnel regarding PHTLS. 

## Methods: 

***Study design and setting***

In this pre- and post-test study, 144 EMS staff members of East Azerbaijan province who were participating in PHTLS courses during 2017 were divided into two groups of LBL and PBL. Both groups received four sessions of PHTLS training based on 8^th^ edition of PHTLS guideline. The participants’ knowledge and skills were evaluated before and three months after training and the two groups were compared in this regard. Informed consent was obtained before administering the questionnaire. The ethics committee of Tabriz University of Medical Sciences approved the study (Ethic No. IR.TBZMED.REC.1396.421). The score of each participant was kept secret and only the organizer of courses knew it. 

***Participants***

All of the paramedics working in Tabriz EMS (154 persons) were included as study population. During courses, 10 participants were excluded from the study due to not completing the sessions or filling the post-test. Participants were divided into two equal groups of 72 cases. First group had the course in LBL method and the second group in PBL method. 

***Data gathering***

Demographic variables (age, employment duration, level of education) were collected using a pre-designed check list and knowledge and skill were measured and registered before and after implementation of the training course using reliable and valid questionnaires, by an emergency medicine resident. 

**Table 1:** Comparing the baseline characteristics of participants between the two learning method groups

**Variable**	**Problem based **	**Lecture based **	**P-value**
**Age (years)**			
Mean ± SD	34.96±8.40	36.60±8.16	0.18
**Employment duration (years)**			
Mean ± SD	7.61±5.63	8.67±6.66	0.24
**Educational degree**			
Bachelor	37 (38.9)	42 (47.7)	0.23
Associate degree	58 (61.1)	46 (52.3)	

Data are presented as mean ± standard deviation (SD) or frequency (%).

**Table 2:** Comparing the knowledge and skills of emergency medicine services (EMS) personnel before and after training with problem based and lecture based learning methods

**Variables**	**Problem based **	**Lecture based **	**P value**
**Knowledge**			
Before	62.56±12.93	64.69±13.93	0.29
After	82.58±4.96	79.48±3.71	<0.001
Change	38.27±32.69	29.77±36.88	0.01
**Skills**			
Before	57.80±18.88	60.00±20.67	0.45
After	116.91±12.85	79.81±20.20	<0.001
Change	121.69±65.03	39.09±21.37	<0.001

Data are presented as mean ± standard deviation.

**Appendix 1:** Checklist for evaluating the participants’ skills on managing trauma patients in the pre-hospital setting

Action		Weight	Done		Not done^
			Appropriate^$^	Inappropriate#	
1	Cervical spine immobilization	10*			
2	Airway management if needed	10*			
3	Oxygen administration	8*			
4	Peripheral IV line establishment	8*			
5	External bleeding control	8*			
6	Spinal هmmobilization	8*			
7	Serum administration	7*			
8	Immobilization of limb fracture	6*			
9	Vital signs	4*			

$: two points; #: one point; ^: zero point

***Intervention***

Knowledge of participants who attended four sessions in August, September, October and November 2017 was measured before (pre-test) as well as three months after the sessions (post-test). Their knowledge was measured using PHTLS 8^th^ edition questionnaire (15).

Participants’ skills on managing trauma patients in the pre-hospital situations were also measured before and three months after PHTLS sessions using a checklist containing criteria of basic and advanced life support, time to response and time to reach the hospital (appendix 1). The checklist was prepared according to expert opinion and based on PHTLS concept and its validity (0.72) and reliability (0.90) were defined using test-re-test method. 

The skills of paramedics were evaluated when treating trauma patients who presented to emergency department of Imam Reza, referral trauma center, Tabriz.

For each EMS staff member, only one multiple trauma patient was selected. In this exam, outcome of the course was pass or fail. Paramedics who failed the exam had to repeat the course in the same manner until they could pass successfully. After 3 months, participants were evaluated in the triage area while they transferred a multiple trauma patient to emergency department. An emergency medicine resident did the evaluation and filled the checklist of evaluation without the EMS personnel sensing. There was not any interference in the standard course of patient treatment. The resident responsible for data gathering was not aware of the type of learning method as well as final scores of participants.

The PHTLS courses were held in Tabriz EMS center. 

We had no loss to follow up because all of the trained paramedics had to refer trauma patients of all around Tabriz to the sole referral hospital of the region.

For preventing bias, the two groups were trained by one faculty member and outcomes (knowledge and skills) were measured by a trained emergency medicine resident.

***Statistical analysis***

All data were analyzed using SPSS software (version 23; SPSS Inc., Chicago, IL). The results are expressed as mean ± standard deviation or percentage. Kolmogorov-Smirnov test was used to assess normal distribution of data. Chi square test, independent T-test or Mann-Whitney U test were used to compare data between the groups. Paired-samples t-test or Wilcoxon rank test were used to compare pre- and post-test results.  P-values less than 0.05 were considered statistically significant. 

## Results:

In this study 144 male EMS staff members were evaluated in two learning groups. The two groups were similar regarding the baseline characteristics (table 1). The mean knowledge score (63.59±13.43 vs 81.08±4.66; p<0.001) and mean skill score (58.85±19.74 vs 99.07±25.02; p<0.001) of participants had significantly improved after applying the training methods. Participants’ scores on knowledge and skills before and after training and their changes are shown in table 2. Both groups had similar knowledge and skills score before intervention, but PBL group had significantly higher scores in both items after intervention. There was also a significantly higher change in knowledge and skills in PBL group compared to LBL group. 

## Discussion:

Our study showed that PHTLS training improves EMS personnel knowledge and skill in managing trauma patients and PBL method was more effective than LBL. Pre-hospital care is a critical phase to improve trauma patients’ outcomes during episodic care. EMS and first-responder personnel must be capable of providing life-saving interventions at scene with proper, quick and correct treatment decisions; so, there is a need to train these personnel to have high confidence and skills in initiating procedures under adverse circumstances (16). 

For this purpose, PHTLS courses have been introduced and used for training paramedics and technicians. Their aim is to increase the skills of EMS personnel in managing trauma patients by improving their knowledge of trauma care and make them able to act faster in life-threatening situations. Cognitive knowledge, technical skills and clinical judgment are the main pillars for healthcare providers (17).

In this study, we evaluated the knowledge and skills of EMS personnel before and after PHTLS courses and observed that overall, there was a significant improvement in participants’ knowledge of the situation, management, and their skills when encountering trauma patients after training. 

Frank and colleagues (10) reported that PHTLS leads to significant increase in confidence and knowledge. Häske et al. (2) reported that the participants’ skills and knowledge on pre-hospital care of trauma patients would increase in short term, but the knowledge and safety would not remain the same on some issues in long term. A systematic review demonstrated improved management of trauma patients and shortened time to definitive care following PHTLS training (18). Improved individual management of trauma patients was also reported following primary trauma care courses (19). Van Dillen and colleagues (16) observed that simulation training improved prehospital care providers’ confidence level in performing two life-saving procedures. Therefore, any type of training would help improve EMS personnel’s confidence and skill, and as previous studies in the same area with our study have suggested, EMS personnel require more education and supervision to provide services according to PHTLS guidelines (20).

Previous studies indicated that the effects of training would decrease after a period (2, 18). Although we did not evaluate the participants in long term, but it is obvious that continuing training programs are necessary for an effective and sufficient EMS, and personnel should attend PHTLS courses at least once a year.

Besides these findings, we evaluated the effects of problem based and LBL methods of training on achieving better knowledge and skills and observed that problem based method led to a significantly greater improvement in participants’ knowledge and most importantly their skills in managing trauma patients. 

PBL enables students to identify the gaps in their knowledge and enhance their group functioning and generic skills (21). Most participants in medical courses prefer practical trainings to theoretical knowledge. Faisal and colleagues (13) have also indicated that PBL is more effective than LBL in the academic performance of medical students. 

***Limitations***

This study was confined by some limitations; being single-centered and having a small sample size were among the main limitations of the study. We did not follow the participants to evaluate the long term efficacy of PHTLS training. In addition, the effect of PHTLS training on reducing time to respond and mortality rate were not evaluated. Although we did not evaluate the long-term efficacy of PHTLS training, continuing training programs are necessary for an effective and sufficient EMS. In further evaluations, the proper time to re-educate the personnel should also be evaluated. We recommend evaluating the personnel in long-term periods, like two and five years, for better results. 

Paramedics who are working in rural pre-hospital centers were not included in this study. It was not possible to gather all of the paramedics working in East Azerbaijan province to participate in courses at the same time. Another limitation is that we could not follow up the maintenance of efficacy of education for more than three months. It would be better if the knowledge and skills of participants were measured 6-12 month later to evaluate the behavior change in management of trauma patients.

## Conclusion:

PHTLS training improves EMS personnel’s knowledge and skill in managing trauma patients. PBL was the more effective method of training compared to traditional LBL. 
